# Case report: Unveiling the unforeseen: a catastrophic encounter of giant aortic aneurysm rupture during re-sternotomy in a patient with bicuspid aortic valve and previous surgical aortic valve replacement

**DOI:** 10.3389/fcvm.2023.1263897

**Published:** 2023-09-13

**Authors:** Hao Niu, Lu Liu, Xiangfeng Gong, Haochen Wang, Yingqiang Guo, Eryong Zhang, Zhenghua Xiao

**Affiliations:** ^1^ Department of Cardiovascular Surgery, West China Hospital, Sichuan University, Chengdu, China; ^2^ Department of Critical Care Medicine, West China Hospital, Sichuan University, Chengdu, China

**Keywords:** giant aortic aneurysm, bicuspid aortic valve, aortic aneurysm rupture and hemorrhage, deep hypothermia circulatory arrest, redo cardiac surgery, case report

## Abstract

Due to structural abnormalities in the leaflets, patients with bicuspid aortic valve (BAV) may develop isolated aortic valve disease, such as aortic regurgitation, aortic stenosis, or a combination of both. In addition to valvular pathology, numerous studies have indicated that approximately 40% of BAV patients exhibit aortic pathologies characterized by aortic dilatation. According to guidelines for valvular diseases, patients with BAV who require surgical aortic valve replacement (SAVR) and have a diameter of the aortic sinuses or ascending aorta ≥4.5 cm are recommended to undergo concomitant replacement of the aortic sinuses or ascending aorta. However, we encountered a case in 2020 involving a patient with severe aortic regurgitation due to BAV and an ascending aortic diameter of 4.2 cm. This patient underwent SAVR and ascending aortoplasty surgery at our center. Remarkably, three years postoperatively, the patient's aortic diameter rapidly expanded by nearly threefold, which also suggests the risk of encountering a giant aortic root aneurysm during reoperation. Unfortunately, a fatal rupture of a giant aortic root aneurysm was encountered during re-sternotomy. Fortunately, with adequate preoperative planning, we successfully managed to avert this perilous situation. The patient recovered without complications and was discharged on the 8th day. Individualized surgical plans were formulated based on a comprehensive evaluation of the perioperative conditions.

## 1. Introduction

BAV is the most prevalent congenital anomaly, affecting 0.5% to 2.0% of adults, with a male-to-female ratio of 3:1. The typical clinical manifestations of BAV include aortic stenosis and regurgitation, but accumulating evidence suggests that approximately 20%–40% of patients also exhibit aortic dilatation (1). This aortopathy can occur independently of valve function and involves the dilation of the aortic sinuses, ascending aorta, or arch. Consequently, patients with BAV require meticulous evaluation of both the aortic valve and the aorta throughout their lifetimes. The progressive dilation of the aorta poses a risk of aortic rupture or dissection, which significantly impacts prognosis (1, 2). The timing and approach for aortic replacement surgery depend on the aortic anatomy, patient characteristics, and institutional expertise. In cases where SAVR is performed due to severe aortic stenosis or regurgitation, concomitant replacement of the ascending aorta is considered appropriate when the aortic diameter exceeds 4.5 cm (2). In this report, we present our experience in managing a male patient with BAV who suffered a rupture of a giant aortic aneurysm during re-sternotomy, three years after the initial SAVR procedure. Our approach involved tailoring an individualized surgical plan based on a comprehensive perioperative assessment. Furthermore, it is important to further explore whether the timing and criteria for concomitant aortic replacement surgery should be more aggressive in BAV patients undergoing SAVR.

## 2. Case presentation

**Figure 1:**
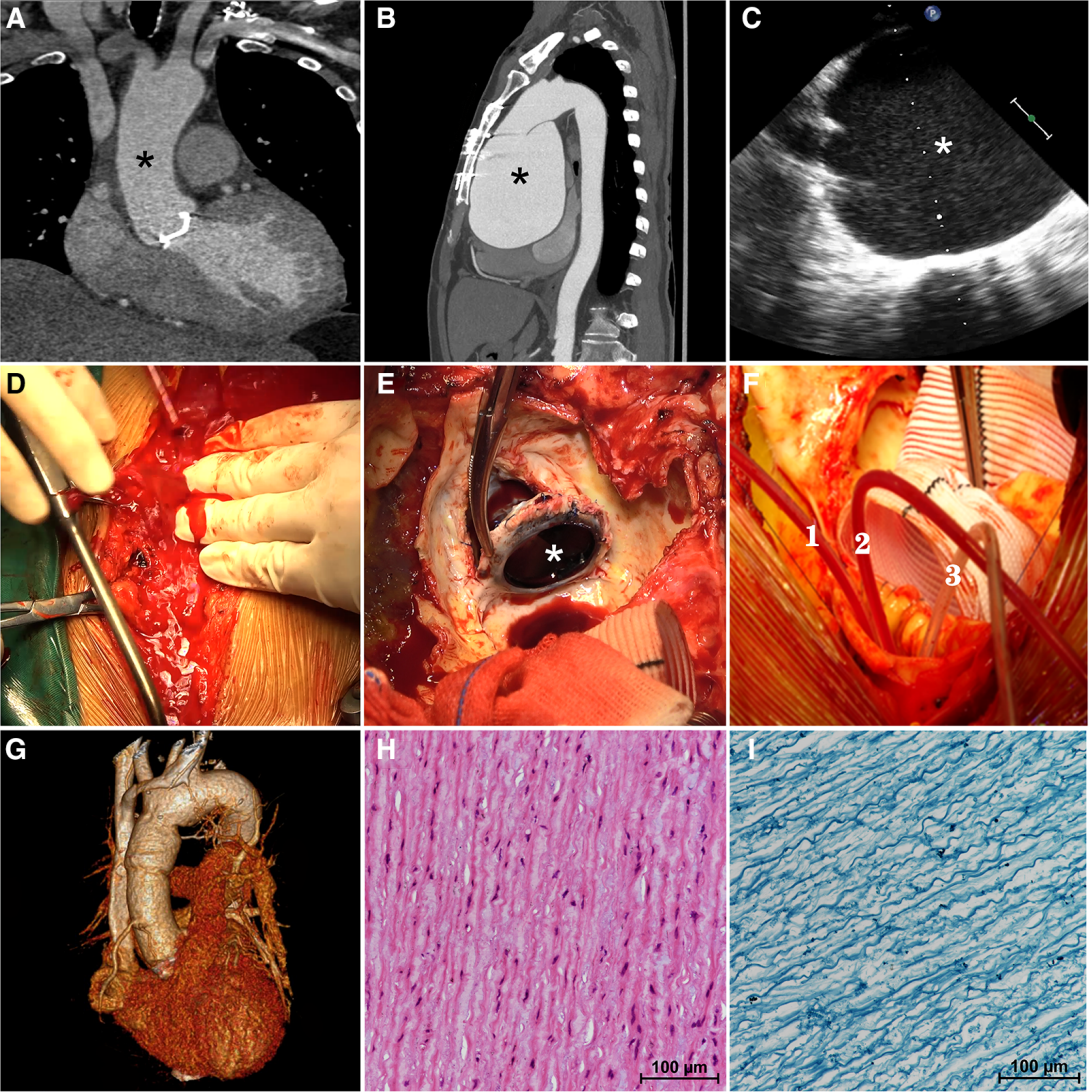
On computerized tomography angiography (CTA) performed at the time of his discharge three years ago, the aortic diameter was measured to be a normal 3.0 cm (**A**, asterisk). The preoperative computerized tomography angiography (**B**) confirmed a giant aortic root aneurysm (asterisk) bulging against the sternum. The transesophageal echocardiography (**C**) confirmed a giant aortic root aneurysm (asterisk) and a functional aortic mechanical valve during the operation. The aortic aneurysm ruptured suddenly during re-sternotomy (**D**) previous mechanical aortic valve (**E**, asterisk) was removed. Three pediatric urinary catheters (**F**, 1.2.3) were used to block the three branches of the aortic arch during hemi-arch replacement. Postoperative three-dimensional enhanced computerized tomography angiography (**G**) showed a normal-diameter aorta as well as relief of compression of the surrounding tissues. Hematoxylin-eosin staining (**H**) and Elastic Verhoeff-Van Gieson's staining (**I**) of aortic aneurysm wall showed the disordered arrangement of smooth-muscle cell and the partial loss of elastin and collagen fibers.

The patient in question is a 57-year-old male who underwent mechanical aortic valve replacement and ascending aortoplasty surgery at our center three years ago due to severe aortic valve regurgitation and ascending aorta dilation (4.2 cm) caused by BAV. At the time of his discharge, a Computerized Tomography Angiography (CTA) was performed, which revealed a normal aortic diameter of 3.0 cm (Figure 1A, asterisk). However, the patient did not return for follow-up examinations at our center after being discharged.

Recently, the patient was admitted to our clinic due to progressively worsening chest tightness and dyspnea, which had persisted for over six months. Upon admission, the physical examination revealed a heart rate of 96 beats/min with sinus rhythm, a blood pressure of 134/90 mmHg, and the presence of a mechanical valve murmur in the aortic valve auscultation area. Importantly, a Transthoracic Echocardiogram (TTE) indicated the presence of a giant aortic root aneurysm measuring 109 mm, with no abnormalities observed in the function of the artificial mechanical aortic valve. The left ventricle (LV) was of normal size (55 mm in end-diastolic dimension), but exhibited a reduced ejection fraction (EF) of 45%. The diagnosis was confirmed by CTA, which showed a protruding giant aortic root aneurysm pressing against the sternum (Figure 1B, asterisk), causing compression of the right atrium and the pulmonary artery.

**Figure 2:**
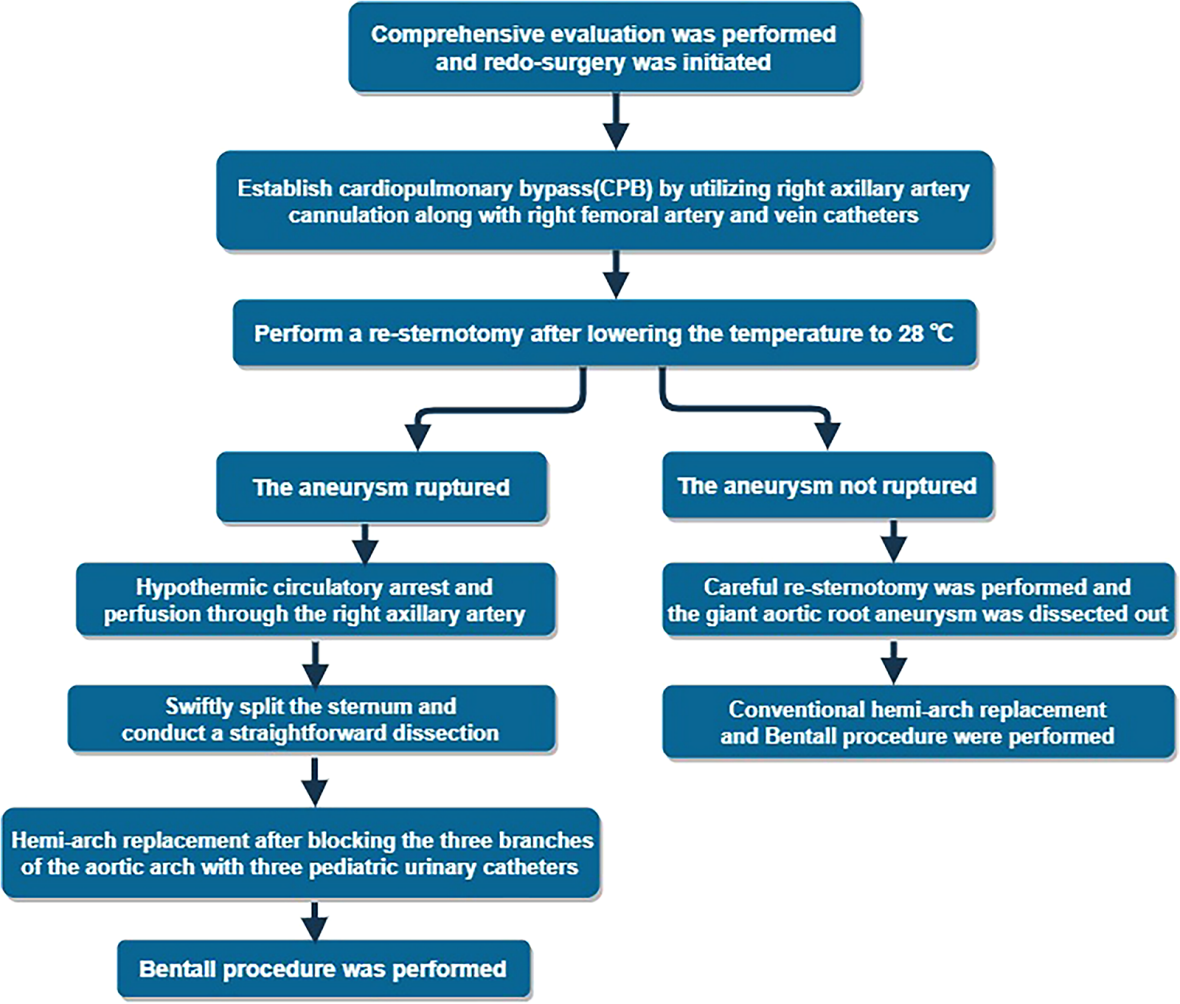
The redo-surgery intervention planning scheme.

After careful multidisciplinary discussions, surgical intervention was deemed necessary and the appropriate life-saving treatment option, considering the risk of rupture/dissection of the aortic root aneurysm and the worsening cardiac function. Upon reopening the sternum in a caudad to cephalad direction, a dense adhesion between the ascending aorta and the sternum was encountered, making it unsafe to perform dissection under high aortic pressure. The surgical plan involved a re-sternotomy procedure with the support of cardiopulmonary bypass (CPB), utilizing right axillary artery cannulation along with right femoral artery and vein catheters. This approach was chosen due to the presence of a massive aneurysm at the aortic root and the potential risk of fatal rupture during the re-sternotomy procedure due to its close proximity to the sternum (Figure 2).

Once the patient was anesthetized, a transesophageal echocardiogram (TEE) confirmed the presence of a giant aortic root aneurysm (Figure 1C, asterisk) along with a functional mechanical aortic valve. Subsequently, right axillary cannulation was performed to establish anterograde cerebral perfusion, and CPB was initiated through the right common femoral artery and vein. Systemic cooling was employed to decompress the aorta and prepare for possible circulatory arrest. CPB was successfully established without any complications following the right axillary artery and elective right femoral arteriovenous cannulation, ensuring the avoidance of unexpected rupture of the giant aneurysm. The patient was cooled systemically, and the sternotomy procedure commenced once the body temperature reached the targeted level of 28°C.

During the carefully prepared sternotomy, the aneurysm unexpectedly ruptured, leading to a sudden rush of bright red arterial blood onto the surface of the sternum (Figure 1D, Supplementary Material, Video S1). Immediate measures were taken, initiating hypothermic circulatory arrest and perfusion through the right axillary artery to prevent cerebral air embolism. Simultaneously, the sternotomy procedure was continued, swiftly splitting the sternum and conducting a straightforward dissection. The ruptured giant aortic root aneurysm nearly filled the pericardial cavity. Subsequently, the patient underwent hemi-arch replacement using a Hemashield Platinum #32 graft after promptly blocking the three branches of the aortic arch with three pediatric urinary catheters (#8) (Figure 1F, 1.2.3, Supplementary Material, Video S2), facilitating the implementation of unilateral antegrade cerebral perfusion via the right axillary artery. The previous mechanical valve was removed (Figure 1E, Supplementary Material, Video S3), and an aortic root replacement with a composite valve graft (CVG) procedure (Bentall) was performed using a 21 mm St. Jude Regent composite and a 28 mm conduit under CPB. The patient was successfully weaned off cardiopulmonary bypass, and the chest was closed using standard procedures. The duration of cardiopulmonary bypass was 196 min.

Following the surgical procedure, the patient was transferred to the intensive care unit while still intubated and was successfully extubated after two days. A three-dimensional CTA performed prior to discharge revealed a normal-sized aortic root with no evidence of pressure on the right atrium and pulmonary artery (Figure 1G). Histological analysis of the aortic aneurysm wall, using Hematoxylin-eosin staining (Figure 1H) and Elastic Verhoeff-Van Gieson's staining (Figure 1I), demonstrated smooth muscle cell disorder and partial loss of elastin and collagen fibers. The patient's aortic graft remained stable without any signs of compression or symptoms of heart failure, and after an eight-day hospital stay, the patient was discharged without any complications. The informed consent for the surgical procedure was obtained from the patient.

## 3. Discussion

We have presented a case of a male patient with BAV who experienced a rupture of a giant aortic aneurysm during re-sternotomy, three years after the initial SAVR procedure. The successful management of this case was achieved through a well-planned perioperative approach. While it is common for the ascending aorta to continue dilating in BAV patients, the nearly threefold dilation observed in this case over a span of three years is highly uncommon, especially after the patient had previously undergone SAVR and ascending aortoplasty surgery.

In patients with BAV who have undergone aortic valve replacement, it is reasonable to conduct lifelong periodic imaging of the aorta if the diameter of the aortic sinuses or ascending aorta is ≥4.0 cm (1). Additionally, annual aortic imaging is advisable for patients with BAV and significant aortic dilation (>4.5 cm) to determine the appropriate timing for surgical intervention (2). Patients with risk factors that increase the likelihood of aortic dissection, such as a rapid rate of change in aortic diameter or a family history of aortic dissection, may require more frequent monitoring (2). However, limited data exist regarding the degree of aortic dilation in BAV patients that would warrant concurrent ascending aortic replacement during SAVR. Several studies have explored the risk of progressive aortic dilation and dissection following SAVR in BAV patients, but definitive conclusions are lacking (2–4).

According to the 2020 ACC/AHA guidelines, when patients undergo SAVR due to severe aortic valve stenosis or regurgitation, replacement of the ascending aorta is considered reasonable if the aortic diameter exceeds 4.5 cm (2). However, in the case we encountered, it is evident that the indications for concomitant ascending aortic replacement during the previous SAVR procedure three years ago should have been more aggressive. Such approach might have potentially prevented the occurrence of this high-risk re-sternotomy event. Although ascending aortoplasty shows good early results in patients with aortic valve disease and dilatation of the ascending aorta and is generally limited to selected patients with high perioperative risks or borderline aortic dilatation. Just like in this case, re-dilatation tends to happen in patients with BAV, and long-term follow-up is necessary.

The development of bicuspid aortopathy can be attributed to abnormal valve dynamics, as even normally functioning bicuspid aortic valves can exhibit abnormal transvalvular flow patterns. These patterns lead to regional increases in wall shear stress, which are predominantly predicted by the morphological features of the bicuspid valve (1). This concept is supported by observations in which the mechanical bileaflet valve replaced three years ago may have had persistent hemodynamic impacts, potentially contributing to the rapid dilation of the aorta following SAVR.

During open arch repair with circulatory arrest, the use of axillary artery cannulation as the arterial access site can help prevent embolic stroke and reduce early mortality (5). Cerebral protection strategies for proximal aortic and arch surgery have undergone significant advancements (6). Deep hypothermic circulatory arrest (DHCA) with antegrade cerebral perfusion (ACP) is a widely utilized method by experienced aortic surgical teams to safeguard the brain by enhancing the safety of circulatory arrest and reducing its metabolic demands (7). However, in patients with previous cardiac operations or complex proximal aortic or transverse arch surgery, the procedure becomes particularly challenging. Such cases often require additional time to complete the repair under circulatory arrest, and the safety of moderate hypothermia is not well established (8). The utilization of hypothermia, selective antegrade cerebral perfusion, and advancements in techniques for proximal and total aortic arch surgery have revolutionized the field of aortic surgery (9).

Re-sternotomy for procedures involving the proximal aorta that require hypothermic circulatory arrest with selective antegrade or retrograde cerebral perfusion is a complex procedure. It is frequently associated with higher morbidity and mortality rates compared to aortic repair performed in the setting of primary sternotomy (10). BAV is characterized by a complex genetic structure involving multiple interacting genes and follows an autosomal dominant inheritance pattern with incomplete penetrance and variable expression (11). The interaction of multiple reciprocal gene deletions contributes to the loss of elastic tissue and collagen fibers during aortic growth (12).

## 4. Conclusion

In conclusion, we have described a rare and complex clinical case involving a male patient with BAV who experienced a rupture of a giant aortic aneurysm during re-sternotomy, three years after the initial SAVR procedure. It can be described as a nightmare scenario. The successful management of this case was achieved through a well-planned perioperative approach. This case underscores the significance of regular follow-up and monitoring of aortic diameter in BAV patients, whether they have undergone valve replacement or not.

It is crucial for clinicians to conduct comprehensive clinical evaluations that take into account factors such as anatomical considerations, cardiovascular risk factors, and coexisting conditions. This holistic evaluation enables the development of a personalized treatment plan that considers the potential costs and risks associated with reoperation. By adopting a patient-specific approach, clinicians can provide optimal care and minimize complications in BAV patients.

Continuous vigilance, regular monitoring, and comprehensive evaluations are essential for the long-term management of BAV patients, particularly in terms of their aortic health. Through meticulous follow-up and proactive intervention, clinicians can improve patient outcomes and mitigate the risks associated with aortic complications in this patient population.

## Data Availability

The original contributions presented in the study are included in the article/**Supplementary Material**, further inquiries can be directed to the corresponding author.

## Supplementary material

SUPPLEMENTARY VIDEO S1

During the redo sternotomy procedure, we encountered a catastrophic rupture and hemorrhage of a massive aortic aneurysm, necessitating an immediate initiation of deep hypothermic circulatory arrest before proceeding with the sternotomy.

SUPPLEMENTARY VIDEO S2

The patient underwent hemi-arch replacement using a Hemashield Platinum #32 graft after promptly blocking the three branches of the aortic arch with three pediatric urinary catheters.

SUPPLEMENTARY VIDEO S3

The previous mechanical valve was removed, and an aortic root replacement with a composite valve graft procedure (Bentall) was performed using a 21-mm St. Jude Regent composite and a 28-mm conduit under CPB.
